# Development and Implementation of a Protocol for In‐Room Surgical Procedures in Paediatric Intensive Care

**DOI:** 10.1111/nicc.70601

**Published:** 2026-07-31

**Authors:** Lucile Revel, Filipa Baptista Peixoto Befecadu

**Affiliations:** ^1^ Department of Neonatology and Pediatric Intensive Care, Department of Women, Children and Adolescents Geneva University Hospitals Geneva Switzerland; ^2^ Research and Implementation Care Lab, Care Directorate Geneva University Hospitals Geneva Switzerland

## Abstract

The Paediatric Intensive Care Unit (PICU) of a Swiss university hospital routinely performs surgical interventions directly in patient rooms due to certain patients' inability to be transferred to the operating room. Given the variability in practice, organizational delays and increased stress among healthcare professionals stemming from oral transmission of procedural knowledge, an institutional protocol was developed. This protocol aims to enhance care safety, improve interdisciplinary coordination and systematically anticipate material human, and environmental needs. Developed through structured methodology including semi‐structured interviews with key stakeholders, it specifically addresses complex procedures such as Extracorporeal Membrane Oxygenation (ECMO) management, thoracic surgeries and postoperative cardiac revisions within intensive care settings. Its implementation is expected to significantly reduce perioperative complications, optimize ergonomics and logistics and improve overall care quality.

## Impact Statements

- What is known about the topic
  ○ Bedside surgery is sometimes required in critically ill children who cannot be safely transferred to the operating room.
  ○ Standardized protocols for bedside surgery in paediatric intensive care units are scarce.
- What this paper adds
  ○ This paper presents a standardized multidisciplinary protocol for bedside surgery in a paediatric intensive care unit.
  ○ The protocol provides a practical framework to improve organization, communication and patient safety during bedside surgical procedures.

## 1 Introduction

For several years, the Paediatric Intensive Care Unit (PICU) of a university hospital has performed surgical procedures directly in patient rooms when the critical condition of the child prevents transfer to the operating room. Between January and March 2025, 14 of such procedures were recorded, including the initiation, revision or removal of an extracorporeal membrane oxygenation (ECMO) device, thoracic closures and postoperative surgical revisions. These procedures are always complex and often urgent. They take place in a non‐standard environment, involving an unstable patient, a team under pressure and human resources that must be adapted in real time. International paediatric ECMO guidelines emphasize that ECMO‐related interventions, particularly in cardiac failure, are highly time‐sensitive and require advanced organizational preparedness, standardized protocols and close interdisciplinary coordination to ensure timely and safe care delivery [1]. While bedside or non‐operating room procedures are increasingly described in critical care settings when patient instability precludes transfer, the literature remains largely focused on technical feasibility and clinical outcomes rather than organizational workflow and team preparation [1, 2, 3, 4, 5]. Existing ECMO guidelines emphasize rapid deployment and interdisciplinary coordination but provide limited operational guidance for structuring complex surgical interventions performed directly in patient rooms. Consequently, there is a need for practical, context‐adapted organizational protocols that translate these general principles into standardized bedside practice [5]. Until now, organizations of surgical procedures directly in patient rooms relied primarily on the oral transmission of knowledge, resulting in heterogeneous practices, organizational delays and significant stress for the professionals involved.

Quality in healthcare is defined as ‘a measure whereby health services for individuals and populations increase the likelihood of desired health outcomes and are consistent with current professional knowledge’ [6]. In high‐risk environments like paediatric intensive care, achieving this level of quality requires clear structures, consistent practices and strong interdisciplinary coordination. In this context, and as highlighted in the literature ‘quality and safety improvements that show the quickest effectiveness seem to be those focusing on an important clinical aspect or a specific clinical process or protocol’, the development of an institutional protocol became essential [7]. This protocol seeks to organize the care environment, anticipate material needs and harmonize practices, thus directly contributing to the delivery of safe, high‐quality and consistent care. The literature also highlights the benefits of clinical protocols in terms of standardizing care, optimizing resource use, strengthening team adherence and improving patient outcomes [8, 9]. Given the low frequency of these procedures and the high turnover of staff, such a tool is essential to reduce variability, secure practices and support healthcare providers in high‐intensity situations.

### 1.1 Project Objective

The objective was to develop and implement a standardized protocol for surgical procedures performed in PICU patient rooms. The protocol aims to improve patient safety, ensure consistent interdisciplinary coordination and anticipate material and organizational needs, thereby reducing variability, delays, and stress among healthcare teams.

## 2 Protocol Development Methodology

The protocol was initiated by the nursing team of the PICU in response to recurrent organizational difficulties observed during in‐room surgical procedures. Grounded in bedside clinical practice, the project emerged from nursing‐identified needs related to patient safety, workflow efficiency and interdisciplinary coordination. It was subsequently developed through a close and continuous collaboration between nursing, medical, surgical and perfusion teams, following a formalized approach to improve nursing practices as recommended in methodological frameworks for clinical protocols [10]. According to Cloarec [11], structuring nursing knowledge into recommendation systems enhances visibility and promotes collective autonomy.

This protocol was developed using a qualitative descriptive and participatory approach involving key stakeholders. Semi‐structured interviews were conducted with the involved healthcare teams to identify needs, define priorities and document barriers encountered during previous in‐room procedures. Interview data were reviewed by the research team to extract key themes related to organizational and procedural challenges. These findings informed the initial protocol draft.

The draft protocol was subsequently iteratively reviewed and tested with all involved stakeholders, including members of the clinical teams. Feedback from these consultations led to several revisions. Finally, the protocol underwent formal validation through the institution's medical and nursing governance process. The operating teams highlighted major issues, particularly systematic delays linked to patient environment preparation. These delays significantly wasted time and complicated optimal patient positioning. Identified needs included improving room ergonomics, installing a dedicated third suction outlet and proactively managing the delivery of an isothermics box for blood pouches. Medical teams from the PICU primarily emphasized coordination difficulties during the preoperative period. Lack of synchronization in professionals' arrival times and variations in anticipatory measures depending on the team members present considerably complicated initial management. These observations underscored the necessity for improved structuring and harmonization of practices. PICU nursing teams highlighted several organizational and human barriers, notably the absence of a formal procedure complicating anticipation of needs. The stress experienced in emergency situations and variability in requests from supervising physicians were also noted, creating misunderstandings and time losses. This medico‐nursing collaborative approach ensured that the protocol was not solely based on theoretical or procedural considerations but firmly anchored in daily clinical reality. Nursing leadership in the project facilitated the translation of clinical needs into practical organizational solutions, while medical input ensured alignment with surgical and intensive care requirements. This shared governance was essential to the protocol's acceptability, feasibility and sustainability in practice.

### 2.1 Ethical Considerations and Governance

Anonymized semi‐structured interviews with healthcare professionals were conducted initially to support the development of the protocol. No patient data were collected, and no modifications to standard patient care were involved. Participation was voluntary, and confidentiality was ensured in accordance with institutional governance requirements. The project was reviewed and approved on December 18, 2024 by the hospital's institutional committee for studies not requiring review by a research ethics committee (*Conseil d'évaluation des études ne relevant pas de la commission d'éthique de la recherche* [CEENCER], protocol approval No. 2024‐23).

## 3 Protocol Content

### 3.1 Objectives and Indications

The protocol primarily aims to secure room‐based interventions through effective anticipation and optimal team coordination. It specifically addresses situations where transfer to the operating room is impossible or inadvisable, such as in cases of haemodynamic instability, pre‐cardiac arrest, life‐threatening emergencies or unavailability of operating room facilities.

### 3.2 Organization and Planning

**FIGURE 1:**
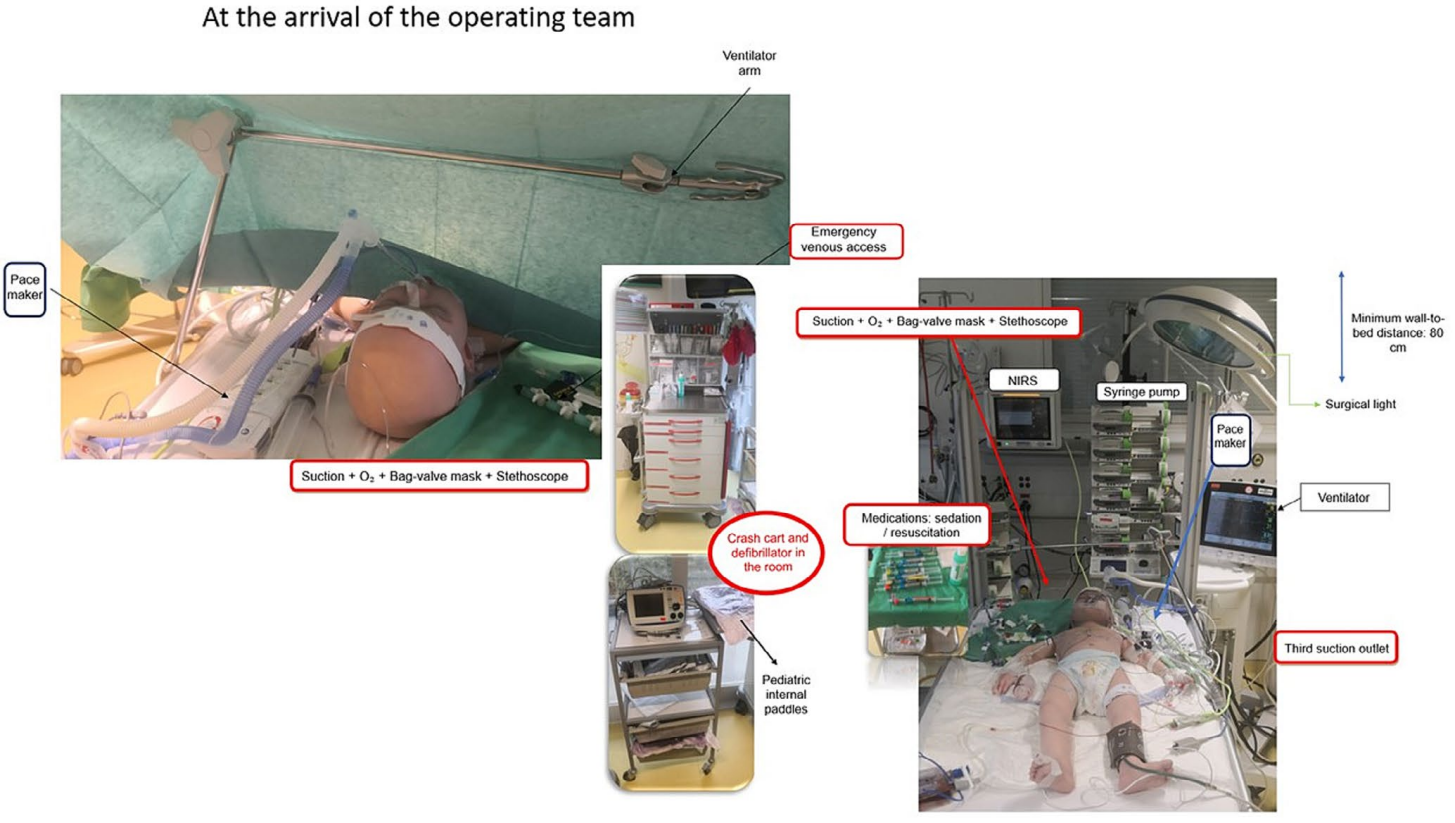
Preoperative environment and patient positioning.

Preoperative preparation is limited to 1 h before the scheduled start of the intervention to optimize human and material resources. The protocol explicitly defines roles for surgeons, perfusionists, instrument nurses and intensive care personnel (nursing assistants, nurses and physicians). It specifies optimal room setup to guarantee sterility and functional ergonomics, as illustrated in Figure 1. A pre‐intervention ‘time‐out’ clearly defines each individual's roles and subsequent steps. Families are informed in advance to manage anxiety and facilitate potential participation.

## 4 Perioperative Risk Management

The protocol addresses major risks associated with room‐based interventions and defines specific preventive measures tailored to each risk category:

- Hypothermia risk: Hypothermia is a significant perioperative risk, especially in neonates. Preventive measures are systematically anticipated: radiant heaters or warming mattresses are installed before the procedure, and survival blankets are used for newborns. These strategies help maintain normothermia and prevent complications such as hemodynamic instability, coagulation disorders.
- Respiratory risks (such as accidental extubation, bronchospasm, and reduced pulmonary compliance): Endotracheal tube security is systematically checked, with continuous capnography and SpO_2_ monitoring. Particular vigilance is required during drape removal to prevent accidental extubation. All patients receive a neuromuscular blockade before skin incision to optimize ventilation, and an experienced ICU nurse monitors pulmonary mechanics in real time.
- Hemodynamic risks (such as blood pressure instability, arrhythmias or intraoperative bleeding): All patients are equipped with an arterial catheter for continuous monitoring, with non‐invasive methods available as backup. Vasoactive and emergency drugs are prepared in advance, and blood products secured in an isothermal box. In specific contexts, such as sternal closure or ECMO decannulation, drug adjustments may be guided by transesophageal echocardiography (TEE).
- Skin and positioning risks (such as pressure ulcers, burns or extravasation): Systematic pressure point checks are performed, with protective devices used to prevent pressure ulcers. Infusion lines are verified to avoid dislodgement or extravasation. Heat‐generating equipment is monitored to prevent burns.
- Pain and agitation: Adequate sedation and analgesia protocols are applied systematically, based on the child's weight, age and clinical condition. A dedicated team member reassesses sedation depth regularly. The medication plan includes pre‐prepared doses for rapid titration in response to any changes.
- Infection control risks (such as surgical site contamination or airborne exposure): As bedside procedures are not performed in an ultra‐clean operating room environment, specific infection prevention measures are applied. Room organization is optimized to limit movement and cross‐traffic during the procedure. A ‘restricted access—procedure in progress’ sign is placed on the door to reduce unnecessary opening and airflow disruption. Only essential staff remain in the room, and students are asked to stay for the entire procedure to avoid repeated entry and exit. Standard aseptic precautions, including sterile draping, surgical attire, and strict hand hygiene, are systematically maintained. These measures are reinforced by a preoperative safety checklist, ongoing interdisciplinary communication and a structured postoperative reassessment, all of which contribute to significantly reducing perioperative complications and ensuring safe, efficient care delivery.

**FIGURE 2:**
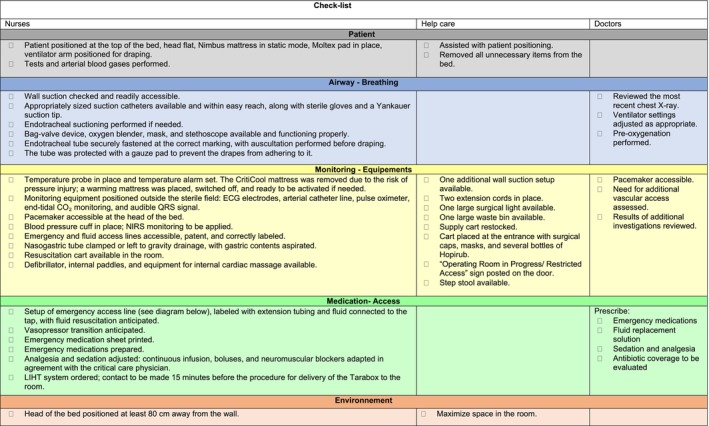
Preoperative safety checklist.

The preoperative safety checklist (Figure 2) helps the multidisciplinary team anticipate and prevent perioperative risks by ensuring that all critical safety measures are completed before the procedure.

### 4.1 Optimizing Ergonomics and Logistics

**FIGURE 3:**
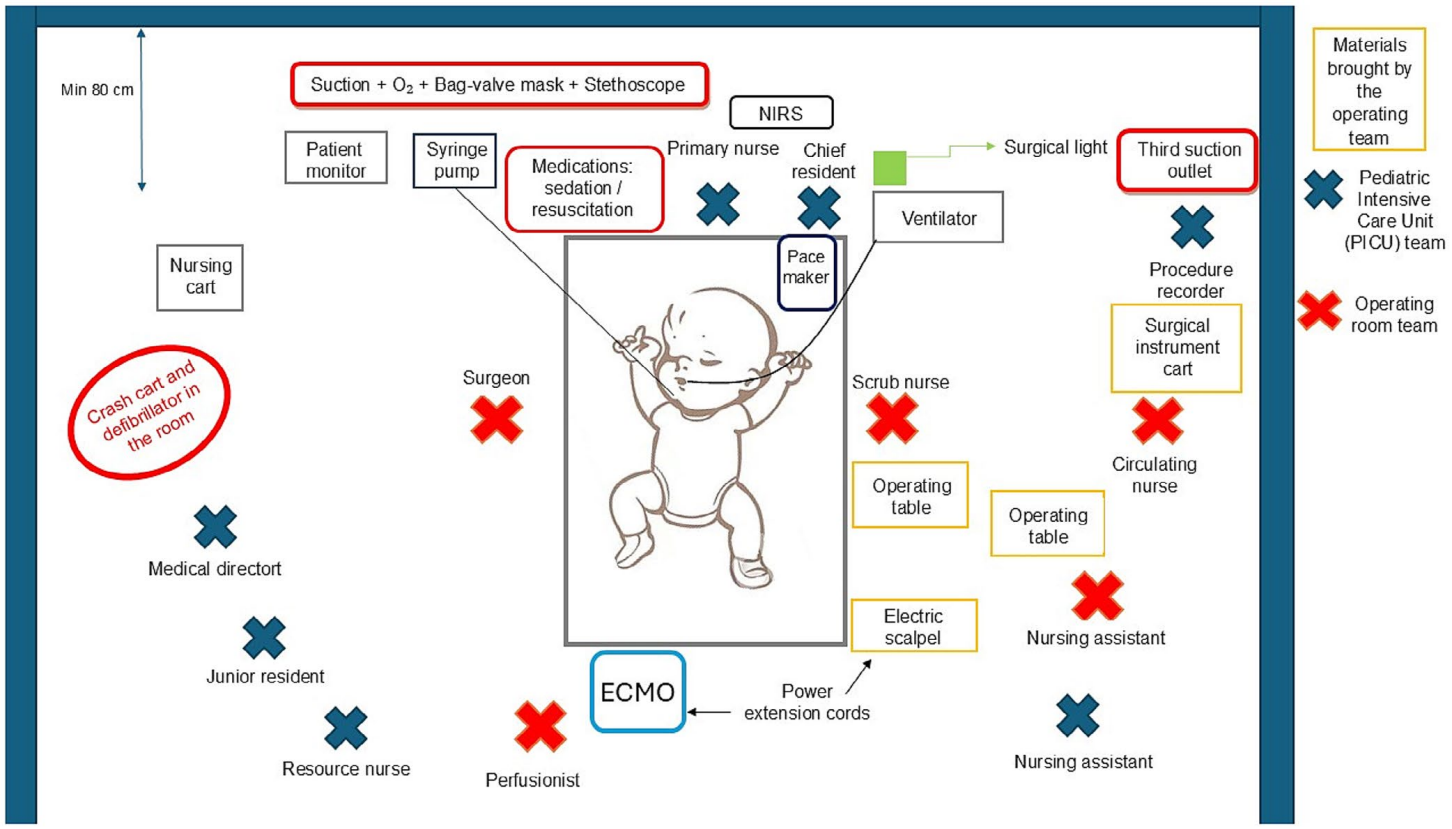
Staff and equipment organization during in‐room surgical interventions.

The protocol emphasizes precise organization of the environment and bed positioning to ensure sufficient space at the patient's head for airway safety while minimizing cross‐actions between teams. Access to essential equipment (monitoring, resuscitation cart, suction devices and surgical lighting) is optimized, and a preoperative checklist ensures functionality and proper placement. Staff and equipment organization during in‐room surgical interventions is illustrated in Figure 3. Close coordination with operating room teams further supports synchronized and efficient interventions.

#### 4.1.1 Medication Management and Perioperative Monitoring

Emergency drugs, as well as agents for sedation, analgesia and neuromuscular blockade, are prepared in advance. Continuous vital sign monitoring allows rapid dose adjustment based on clinical needs.

#### 4.1.2 Postoperative Procedure and Follow‐Up

As during the intervention, monitoring continues in a structured manner using the Temperature, Airway, Breathing, Circulation, Disability and Exposure (TABCDE) approach, with the addition of ‘T’ for Temperature reflecting the importance of thermoregulation in paediatrics [11]. The ABCDE method [12] remains a reference for improving safety, allowing early identification of clinical deterioration and effective response. The team repositions the patient, checks the equipment and reassesses ventilation, sedation and treatments to adapt care. Parents are promptly informed and reunited with their child, while rigorous record updates ensure full traceability of care.

#### 4.1.3 Expected Impact and Improvement Prospects

Implementing this protocol is expected to significantly enhance care safety and efficiency by notably reducing complications, strengthening interdisciplinary coordination and substantially decreasing stress among teams. Realistic simulations involving all stakeholders validated and refined the protocol. Integrated into institutional guidelines, it is regularly communicated and continuously taught to concerned staff. Potential implementation barriers (resource shortages, training needs, resistance to change) have been identified and proactively managed according to Gerling's recommendations [13].

### 4.2 Perspectives

Finally, monthly questionnaires for involved nurses will continuously evaluate protocol utility and identify potential improvements. The study was conducted from December 2024 to December 2025, fully validating the protocol's effectiveness and continuously refining the recommendations to optimize the quality of care.

## 5 Conclusion

The formalization of a specific protocol for surgical interventions performed in PICU patient rooms at HUG addresses a critical need for improving nursing and medical practices in a complex setting. By rigorously structuring patient management, the protocol aims to enhance risk anticipation, optimize material and human resources and standardize interventions. If regularly evaluated and institutionally integrated, it could represent a significant step forward in improving the safety and efficiency of paediatric intensive care, while potentially reducing staff stress and improving clinical outcomes. The results of the evaluation will be published this year.

## Funding

The authors have nothing to report.

## Ethics Statement

Approved by the CEENCER (Council for the Evaluation of Studies Not Requiring Ethics Committee Review) on December 18, 2024 (reference number: 2024‐23).

## Conflicts of Interest

The authors declare no conflicts of interest.

## Data Availability

Data sharing not applicable to this article as no datasets were generated or analysed during the current study.
